# HIV Infection and HIV-Associated Behaviors Among Persons Who Inject Drugs — 20 Cities, United States, 2012

**Published:** 2015-03-20

**Authors:** Michael W. Spiller, Dita Broz, Cyprian Wejnert, Lina Nerlander, Gabriela Paz-Bailey

**Affiliations:** 1 Division of HIV/AIDS Prevention, National Center for HIV/AIDS, Viral Hepatitis, STD, and TB Prevention, CDC

In the United States, an estimated 7% of new diagnoses of human immunodeficiency virus (HIV) infection in 2012 were attributed to injection drug use, and an additional 3% to male-to-male sexual contact and injection drug use (1). To monitor HIV prevalence and behaviors associated with HIV risk and prevention among persons who inject drugs (PWID), CDC’s National HIV Behavioral Surveillance (NHBS) system conducts interviews and HIV testing in selected cities. This report summarizes HIV prevalence and behaviors among PWID interviewed and tested in 20 cities in 2012. Of the 10,002 PWID tested, 11% had a positive HIV test result. Among 9,425 PWID included in the behavioral analysis, 30% receptively shared syringes, 70% had vaginal sex without a condom, 25% had heterosexual anal sex without a condom, and 5% of males had male-to-male sexual contact without a condom in the previous 12 months. Fifty-one percent of PWID included in the behavioral analysis had been tested for HIV, 25% participated in an HIV behavioral intervention, and 39% participated in substance abuse treatment in the previous 12 months. Additional efforts are needed to reduce risk behaviors and increase access to HIV testing, drug treatment, and other HIV prevention programs to further reduce HIV infections among PWID.

In 2012, NHBS staff collected cross-sectional behavioral survey data and conducted HIV testing among PWID recruited using respondent-driven sampling* (2–4) in 20 cities† with high prevalence of acquired immunodeficiency syndrome (AIDS). Persons who volunteered to participate, were eligible§, and consented were administered a standardized, anonymous questionnaire face-to-face by trained interviewers. All participants were offered anonymous HIV testing, performed by collecting blood or oral specimens for either rapid testing in the field or laboratory-based testing. A nonreactive screening test result was considered HIV-negative; a reactive screening test result was considered HIV-positive if confirmed by Western blot or indirect immunofluorescence assay. Incentives were offered for interview completion, HIV testing, and recruitment.¶

PWID with HIV-positive test results during the survey were defined as aware of their HIV infection if they reported a previous HIV-positive test result. Because studies have found that knowledge of personal HIV status might influence risk behaviors (5), behavioral analysis was limited to participants who did not report a previous HIV-positive test result. Participants were asked whether they had, in the 12 months before the interview, engaged in high-risk injection or sex behaviors, been tested for HIV infection, or participated in an HIV behavioral intervention.** In addition, participants were asked whether they had ever been tested for HIV or hepatitis C virus (HCV) infection.†† Data from each city were analyzed using a respondent-driven sampling analysis tool that adjusted for differences in peer recruitment patterns and PWID network size and estimated 95% confidence intervals (CIs) using the Salganik bootstrap variance estimator (6). City-level analyses were aggregated and weighted by the estimated size of the PWID population in each city (7) to obtain estimates overall.§§

In 2012, a total of 13,093 persons were recruited to participate; of these, 2,812 (21%) were ineligible. An additional 279 (2%) eligible participants were excluded from analysis.¶¶ Data for the remaining 10,002 participants were used in the analysis of HIV prevalence (Table 1).

Among 10,002 PWID, 11% (CI = 9%–12%) tested positive for HIV. The percentage of PWID with HIV infection was higher among non-Hispanic blacks (16%) (CI = 13%–18%) than non-Hispanic whites (5%) (CI = 3%–7%). PWID in the South U.S. Census region had higher HIV prevalence (13%) (CI = 11%–16%) than those in the Midwest (8%) (CI = 5%–11%) and West regions (7%) (CI = 5%–10%). The prevalence of HIV infection was lower among PWID who most frequently inject heroin only (7%) (CI = 6%–9%) than among PWID who most frequently inject drugs other than heroin or multiple drugs (17%) (CI = 13%–21%). Prevalence of HIV infection was 27% (CI = 20%–33%) among male PWID who reported male-to-male sex in the previous 12 months. Among HIV-positive PWID, 63% (CI = 55%–70%) were aware of their infection.

Among the 9,425 PWID included in behavioral analysis, 30% (CI = 28%–32%) receptively shared syringes, 70% (CI = 68%–72%) had vaginal sex without a condom, 25% (CI = 23%–27%) had heterosexual anal sex without a condom, and 49% (CI = 47%–51%) had more than one opposite sex partner in the previous 12 months (Table 2). The percentages of PWID who receptively shared injection equipment or had more than one opposite sex partner in the previous 12 months were highest among PWID aged 18–29 years. Among male PWID, 10% (CI = 8%–11%) reported male-to-male sexual contact, and 5% (CI = 4%–6%) reported male-to-male sexual contact without a condom in the previous 12 months.

In addition, 25% (CI = 23%–27%) of PWID participated in an HIV behavioral intervention, 39% (CI = 36%–41%) participated in drug treatment, and 51% (CI = 49%–54%) had an HIV test in the previous 12 months (Table 3). Ever being tested for HCV was reported by 78% (CI = 76%–80%) of PWID.

PWID with health insurance were more likely to have been tested for HIV in the previous 12 months (55%) (CI = 53%–58%) than were PWID without health insurance (44%) (CI = 41%–47%) (Table 3).*** Similarly, more PWID with health insurance reported having participated in an HIV behavioral intervention (27%) (CI = 24%–30%) or having ever been tested for HCV (81%) (CI = 79%–84%) than did PWID without health insurance (HIV behavioral intervention: 20% [CI = 17%–22%]; HCV test: 73% [CI = 70%–76%]).

The change in the percentage of PWID with HIV infection from 2009 (9%) to 2012 (11%) was not statistically significant. Among PWID with HIV-positive test results in 2012, 63% were aware of their infection, which is not significantly different from that found in 2009 (55%) (4). The percentages of PWID who engaged in risk behaviors in 2012 also were consistent with 2009 data (4).

## Discussion

The 2012 data in this report provide updated estimates of the prevalence of HIV infection and behaviors since the last NHBS survey of PWID in 2009 (4). The change in the percentage of PWID with HIV infection from 2009 to 2012 was not statistically significant. The percentage of PWID with HIV-positive test results who were aware of their infection in 2012 also was not significantly different from that found in 2009 (4).

The percentages of PWID who engaged in risk behaviors in 2012 are consistent with 2009 data (4). These percentages highlight a role for expanded HIV testing and prevention among PWID. The high-risk behaviors observed among PWID represent an opportunity to prevent future increases in HIV infections caused by sharing injection equipment or having sex without a condom.

Compared with the last NHBS survey of PWID in 2009, higher percentages of participants in this 2012 study reported participating in HIV behavioral interventions in the previous 12 months (25% in 2012 compared with 19% in 2009) and having ever been tested for HCV infection (78% and 72%, respectively) (4). Similar percentages of PWID reported being tested for HIV in the previous 12 months (51% and 49%, respectively).

This analysis found that PWID with health insurance were more likely to have been tested for HIV infection in the previous 12 months, to have participated in an HIV behavioral intervention in the previous 12 months, and to have ever been tested for HCV than were PWID without health insurance. These differences suggest that expanding health insurance coverage might allow more PWID to become aware of their HIV and HCV status and to have access to important treatment and prevention interventions.

Consistent with previous reports (4,8), this analysis found that younger PWID were more likely to have shared injection equipment or have had more than one opposite sex partner in the previous 12 months than were older PWID. The percentages of PWID who were tested for HIV infection or participated in an HIV behavioral intervention were similar among younger and older PWID.

The findings in this report are subject to at least four limitations. First, some participants might not have accurately reported their behavior to interviewers, and results might be affected by social desirability bias. Second, because no method of obtaining standard probability samples of PWID exists, the representativeness of the NHBS sample cannot be determined. Although respondent-driven sampling adjusts for some sampling biases (2), biases related to participants’ recruitment behavior or their willingness and ability to participate in the interview might have affected the sample. Third, the numbers of participants in some cities were insufficient to permit every estimate to be made in every city. Finally, PWID were interviewed in 20 cities with high AIDS prevalence; findings from these cities might not be generalizable to other cities or states.

To reduce the number of new HIV infections, the National HIV/AIDS Strategy††† calls for intensifying prevention efforts in communities where HIV is most heavily concentrated. At the center of any response to HIV among PWID is a comprehensive, multifaceted prevention strategy, which includes access to sterile injection and drug preparation equipment; treatment for substance use and mental disorders; opioid substitution therapy; counseling, testing, and treatment for HIV infection; education on drug-related and sex-related risks and risk-reduction for PWID and their sex partners; and preexposure prophylaxis for adult PWID at substantial risk for HIV acquisition (9,10). An effective prevention approach for PWID also includes prevention and treatment of other infections, including HCV; thus, integration of multiple service programs for PWID might increase the effectiveness of HIV prevention efforts (9).

What is already known on this topic?

Persons who inject drugs (PWID) in the United States are at increased risk for acquiring human immunodeficiency virus (HIV) infection. In 2009, the National HIV Behavioral Surveillance (NHBS) system, which uses respondent-driven sampling to interview and test for HIV infection PWID living in 20 large cities, found an overall HIV prevalence of 9%.

What is added by this report?

The NHBS in 2012 found an HIV prevalence of 11% (95% confidence interval = 9%–12%) among PWID; of those, 63% had been previously aware of their infection, compared with 55% in 2009, not a statistically significant difference. Among PWID reporting negative or unknown HIV status in 2012, 30% reported sharing syringes, and 70% reported having vaginal sex without a condom in the previous 12 months.

What are the implications for public health practice?

Many PWID are at risk for acquiring HIV infection because of their drug use practices and sexual behaviors, but more than one third of HIV-positive PWID in urban areas with high HIV prevalence were unaware of their infection. Additionally, three quarters of PWID had not participated in an HIV behavioral intervention in the previous 12 months. To prevent infections, PWID need ready access to sterile injection and drug preparation equipment; treatment for substance use and mental disorders; opioid substitution therapy; counseling, testing, and treatment for HIV infection; education on drug-related and sex-related risks and risk-reduction; and preexposure prophylaxis if they are adults and at substantial risk for acquiring HIV infection.

## Figures and Tables

**TABLE 1:** Estimated prevalence of HIV infection among persons who inject drugs (PWID), by selected characteristics — National HIV Behavioral Surveillance System, United States, 2012

Characteristic	Overall*		HIV prevalence*	
	
	%	(95% CI)	%	(95% CI)
**Overall (N = 10,002)**	**100**	**—**	**11**	**(9–12)**
**Sex**				
Men	68	(66–70)	10	(4–16)
Women	32	(30–34)	12	(9–15)
**Race/Ethnicity**				
Hispanic†	24	(22–26)	11	(6–15)
Black, non-Hispanic	41	(39–43)	16	(13–18)
White, non-Hispanic	30	(28–32)	5	(3–7)
Other§	5	(4–6)	—¶	—¶
**Age group (yrs)**				
18–29	13	(11–15)	1	(0–8)
30–39	18	(17–20)	6	(4–8)
40–49	27	(25–29)	18	(14–21)
≥50	42	(40–44)	11	(9–13)
**Education**				
Less than high school diploma	34	(32–36)	13	(11–16)
High school diploma	40	(38–42)	9	(7–12)
More than high school diploma	26	(24–28)	10	(6–13)
**Health insurance**				
Yes	69	(68–71)	13	(11–16)
No	31	(29–32)	5	(3–6)
**Poverty level** **				
At or below federal poverty level	79	(77–80)	12	(10–14)
Above federal poverty level	21	(20–23)	7	(4–9)
**Drug injected most frequently**				
Heroin only	67	(65–69)	7	(6–9)
Other/Multiple††	33	(31–35)	17	(13–21)
**Male-male sex (among males only)**				
Yes	12	(10–14)	27	(20–33)
No	88	(86–90)	8	(2–14)
**Region** §§				
Northeast	37	(24–51)	11	(7–15)
South	29	(15–42)	13	(11–16)
Midwest	8	(0–22)	8	(5–11)
West	24	(10–37)	7	(5–10)

**Abbreviations:** HIV = human immunodeficiency virus; CI = confidence interval.

* Percentages were weighted to adjust for differences in recruitment, the size of participant PWID peer networks, and the size of the PWID population in each city.

† Persons of Hispanic ethnicity might be of any race or combination of races.

§ Includes American Indian/Alaska Natives, Asians, Native Hawaiian or other Pacific Islanders, and persons of multiple races.

¶ Insufficient data.

** Poverty level is based on household income and household size.

†† Other drugs injected alone or two or more drugs injected with the same frequency.

§§ Northeast region includes the cities Boston, Massachusetts; Nassau-Suffolk, New York; New York, New York; Newark, New Jersey; and Philadelphia, Pennsylvania. South region includes Atlanta, Georgia; Baltimore, Maryland; Dallas, Texas; Houston, Texas; Miami, Florida; New Orleans, Louisiana; and Washington, DC. Midwest region includes Chicago, Illinois and Detroit, Michigan. West region includes Denver, Colorado; Los Angeles, California; San Diego, California; San Francisco, California; and Seattle, Washington. San Juan, Puerto Rico, was not included.

**TABLE 2:** Estimated percentage* of persons who inject drugs (PWID) who reported HIV-negative or unknown status and who engaged in behaviors† associated with HIV infection in the previous 12 months, by selected characteristics — National HIV Behavioral Surveillance System, United States, 2012

Characteristic	Receptive syringe sharing		Receptive injection equipment sharing		Had vaginal sex		Had condomless vaginal sex		Had heterosexual anal sex		Had condomless heterosexual anal sex		Had condomless heterosexual sex or receptive syringe sharing		Had more than one opposite sex partner	
							
	%	(95% CI)	%	(95% CI)	%	(95% CI)	%	(95% CI)	%	(95% CI)	%	(95% CI)	%	(95% CI)	%	(95% CI)
**Overall (N = 9,425)**	**30**	**(28–32)**	**55**	**(53–57)**	**82**	**(80–83)**	**70**	**(68–72)**	**31**	**(29–33)**	**25**	**(23–27)**	**77**	**(75–79)**	**49**	**(47–51)**
**Sex**																
Men	29	(27–31)	54	(51–56)	82	(80–84)	69	(67–72)	32	(30–34)	26	(24–29)	76	(74–78)	51	(48–53)
Women	34	(30–38)	57	(53–61)	80	(77–84)	72	(68–76)	28	(24–32)	22	(19–26)	79	(76–82)	46	(42–50)
**Race/Ethnicity**																
Hispanic§	32	(28–36)	53	(47–58)	86	(83–89)	73	(69–78)	40	(34–45)	34	(29–39)	81	(76–85)	48	(43–53)
Black, non-Hispanic	21	(18–23)	48	(45–51)	81	(78–83)	67	(64–70)	26	(24–29)	21	(18–23)	73	(70–76)	50	(47–53)
White, non-Hispanic	42	(37–46)	67	(63–71)	79	(76–83)	72	(68–76)	30	(27–34)	25	(21–28)	80	(76–84)	51	(47–55)
Other¶	29	(20–38)	53	(42–65)	82	(75–89)	73	(65–81)	27	(19–35)	22	(15–30)	81	(73–88)	48	(39–56)
**Age group (yrs)**																
18–29	40	(32–47)	73	(67–79)	94	(90–98)	86	(81–92)	43	(36–51)	38	(31–45)	91	(86–97)	68	(61–74)
30–39	40	(36–44)	61	(55–66)	90	(87–93)	80	(76–85)	40	(34–45)	31	(27–36)	85	(81–89)	56	(51–62)
40–49	31	(28–35)	57	(53–60)	82	(78–85)	70	(67–74)	33	(29–36)	27	(24–31)	79	(76–82)	48	(44–51)
≥50	23	(21–26)	47	(44–50)	73	(70–76)	60	(57–63)	22	(20–25)	17	(15–20)	68	(65–71)	42	(39–45)
**Education**																
Less than high school diploma	33	(30–36)	56	(52–60)	83	(80–85)	71	(68–74)	32	(29–35)	27	(23–30)	79	(77–82)	49	(46–53)
High school diploma	31	(28–34)	56	(52–59)	84	(81–87)	72	(69–75)	32	(29–36)	27	(24–30)	78	(75–81)	52	(48–55)
More than high school diploma	27	(23–30)	56	(52–60)	77	(74–81)	67	(63–71)	27	(24–31)	21	(18–24)	74	(70–77)	45	(41–49)
**Health insurance**																
Yes	29	(27–32)	54	(52–57)	80	(78–83)	68	(66–71)	30	(27–33)	25	(22–28)	75	(73–78)	46	(43–49)
No	32	(29–35)	56	(52–59)	84	(82–87)	76	(73–78)	32	(29–35)	26	(23–29)	81	(79–84)	55	(52–58)
**Poverty level** **																
At or below federal poverty level	31	(29–33)	54	(52–57)	81	(79–83)	70	(68–72)	31	(29–33)	26	(23–28)	77	(75–79)	49	(47–52)
Above federal poverty level	29	(25–33)	56	(52–60)	82	(78–85)	70	(66–74)	30	(26–34)	23	(20–27)	76	(72–80)	48	(44–53)
**Drug injected most frequently**																
Heroin only	30	(28–32)	54	(51–56)	82	(80–84)	71	(69–73)	29	(27–32)	24	(22–27)	79	(77–81)	46	(43–49)
Other/Multiple††	31	(28–34)	57	(53–61)	83	(80–86)	70	(67–74)	36	(32–39)	28	(24–31)	78	(74–82)	58	(54–62)
**Region** §§																
Northeast	34	(30–37)	55	(50–59)	85	(82–88)	74	(70–78)	37	(33–41)	31	(26–35)	80	(77–84)	52	(48–57)
South	28	(25–31)	56	(52–59)	82	(79–85)	68	(65–71)	28	(25–32)	23	(20–26)	75	(72–78)	52	(48–55)
Midwest	20	(14–25)	45	(39–51)	83	(78–88)	73	(67–78)	20	(15–25)	17	(12–22)	75	(70–81)	42	(35–48)
West	32	(28–36)	57	(53–61)	76	(73–79)	67	(64–71)	26	(23–30)	22	(19–26)	75	(72–78)	43	(39–47)

**Abbreviations:** HIV = human immunodeficiency virus; CI = confidence interval.

* Percentages were weighted to adjust for differences in recruitment, the size of participant PWID peer networks, and the size of the PWID population in each city.

† Receptive sharing of syringes was defined as "using needles that someone else had already injected with," and receptive sharing of injection equipment was defined as using equipment such as cookers, cottons, or water used to rinse needles or prepare drugs "that someone else had already used." Condomless vaginal sex/Condomless anal sex was defined as "sex without a condom."

§ Persons of Hispanic ethnicity might be of any race or combination of races.

¶ Includes American Indian/Alaska Natives, Asians, Native Hawaiian or other Pacific Islanders, and persons of multiple races.

** Poverty level is based on household income and household size.

†† Other drugs injected alone or two or more drugs injected with the same frequency.

§§ Northeast region includes the cities Boston, Massachusetts; Nassau-Suffolk, New York; New York, New York; Newark, New Jersey; and Philadelphia, Pennsylvania. South region includes Atlanta, Georgia; Baltimore, Maryland; Dallas, Texas; Houston, Texas; Miami, Florida; New Orleans, Louisiana; and Washington, DC. Midwest region includes Chicago, Illinois and Detroit, Michigan. West region includes Denver, Colorado; Los Angeles, California; San Diego, California; San Francisco, California; and Seattle, Washington. San Juan, Puerto Rico, was not included.

**TABLE 3:** Estimated percentage* of persons who inject drugs (PWID) who reported HIV-negative or unknown status and who received testing and prevention services, by selected characteristics — National HIV Behavioral Surveillance System, United States, 2012

Characteristics	Was tested for HIV infection in the previous 12 months		Participated in HIV behavioral interventions in the previous 12 months†		Was ever tested for hepatitis C§	
		
	%	(95% CI)	%	(95% CI)	%	(95% CI)
**Overall (N = 9,425)**	**51**	**(49–54)**	**25**	**(23–27)**	**78**	**(76–80)**
**Sex**						
Men	50	(47–53)	24	(22–26)	78	(76–80)
Women	55	(51–58)	26	(22–30)	79	(76–82)
**Race/Ethnicity**						
Hispanic¶	56	(51–61)	26	(21–31)	79	(74–84)
Black, non-Hispanic	54	(51–57)	23	(21–26)	76	(74–79)
White, non-Hispanic	45	(41–49)	24	(20–27)	83	(80–86)
Other**	47	(38–57)	31	(20–42)	85	(78–91)
**Age group (yrs)**						
18–29	54	(47–61)	25	(20–31)	76	(71–82)
30–39	58	(54–62)	27	(22–32)	79	(75–83)
40–49	56	(52–59)	28	(24–32)	79	(76–82)
≥50	48	(45–51)	22	(19–24)	79	(77–82)
**Education**						
Less than high school diploma	52	(49–56)	25	(21–28)	77	(74–80)
High school diploma	51	(47–54)	23	(20–26)	77	(74–80)
More than high school diploma	52	(48–56)	26	(22–30)	82	(79–85)
**Health insurance**						
Yes	55	(53–58)	27	(24–30)	81	(79–84)
No	44	(41–47)	20	(17–22)	73	(70–76)
**Poverty level** ††						
At or below federal poverty level	51	(49–54)	25	(22–27)	78	(75–80)
Above federal poverty level	51	(47–55)	25	(21–28)	82	(79–85)
**Drug injected most frequently**						
Heroin only	51	(48–53)	25	(22–27)	80	(77–82)
Other/Multiple§§	55	(51–58)	25	(22–29)	76	(72–80)
**Region** ¶¶						
Northeast	54	(49–58)	27	(23–31)	80	(76–84)
South	55	(52–58)	21	(18–24)	75	(72–78)
Midwest	48	(42–54)	28	(23–34)	76	(70–82)
West	45	(41–49)	24	(20–28)	82	(78–85)

**Abbreviations:** HIV = human immunodeficiency virus; CI = confidence interval.

* Percentages were weighted to adjust for differences in recruitment, the size of participant PWID peer networks, and the size of the PWID population in each city.

† Participating in an individual or group HIV behavioral intervention (e.g., a one-on-one conversation with a counselor or an organized discussion regarding HIV prevention) did not include counseling received as part of an HIV test or conversations with friends.

§ Testing for hepatitis C virus infection was measured as ever tested or ever received a diagnosis of hepatitis C. All other behaviors are reported for the previous 12 months.

¶ Persons of Hispanic ethnicity might be of any race or combination of races.

** Includes American Indian/Alaska Natives, Asians, Native Hawaiian or other Pacific Islanders, and persons of multiple races.

†† Poverty level is based on household income and household size.

§§ Other drugs injected alone or two or more drugs injected with the same frequency.

¶¶ Northeast region includes the cities Boston, Massachusetts; Nassau-Suffolk, New York; New York, New York; Newark, New Jersey; and Philadelphia, Pennsylvania. South region includes Atlanta, Georgia; Baltimore, Maryland; Dallas, Texas; Houston, Texas; Miami, Florida; New Orleans, Louisiana; and Washington, DC. Midwest region includes Chicago, Illinois and Detroit, Michigan. West region includes Denver, Colorado; Los Angeles, California; San Diego, California; San Francisco, California; and Seattle, Washington. San Juan, Puerto Rico, was not included.

## Footnotes

* Recruitment chains in each city began with three to 15 initial participants identified during formative assessment. Initial participants who completed the interview were asked to recruit up to five other PWID using a coded coupon system designed to track referrals. Referred and surveyed PWID were also asked to recruit up to five other PWID.

† State and local health departments eligible to participate in NHBS are among those whose jurisdictions include a metropolitan statistical area (MSA) or a specified division with high burden of acquired immunodeficiency syndrome. In 2012, NHBS was conducted in 20 MSAs/divisions. Throughout this report, MSAs and divisions are referred to as cities. The 20 cities were Atlanta, Georgia; Baltimore, Maryland; Boston, Massachusetts; Chicago, Illinois; Dallas, Texas; Denver, Colorado; Detroit, Michigan; Houston, Texas; Los Angeles, California; Miami, Florida; Nassau-Suffolk, New York; New Orleans, Louisiana; New York, New York; Newark, New Jersey; Philadelphia, Pennsylvania; San Diego, California; San Francisco, California; San Juan, Puerto Rico; Seattle, Washington; and Washington, District of Columbia.

§ Persons were eligible to participate if they had injected drugs during the previous 12 months, resided in the city, were aged ≥18 years, and could complete the interview in English or Spanish.

¶ The incentive format (cash or gift card) and amount varied by city based on formative assessment and local policy. A typical format included $25 for completing the interview, $25 for providing a specimen for HIV testing, and $10 for each successful recruitment (maximum of five).

** Receptive sharing of syringes was defined as “using needles that someone else had already injected with,” and receptive sharing of injection equipment was defined as using equipment such as cookers, cottons, or water used to rinse needles or prepare drugs “that someone else had already used.” Condomless vaginal sex/condomless anal sex was defined as “sex without a condom.” Persons tested for HIV infection include those with results that were negative, indeterminate, or unknown. Participating in an individual or group HIV behavioral intervention (e.g., a one-on-one conversation with a counselor or an organized discussion regarding HIV prevention) did not include counseling received as part of an HIV test or conversations with friends. Male-to-male anal sexual contact was restricted to males and included both insertive and receptive anal sexual contact.

†† Testing for HCV infection was measured as ever tested or ever received a diagnosis of hepatitis C.

§§ For city-level estimates for which CIs could not be calculated, maximally wide CIs (0–1) were used in aggregation. City-level estimates with insufficient data for analysis were excluded from aggregation. Such estimates represented 4% of the estimates included in this analysis.

¶¶ Data from 279 participants were excluded because of missing recruitment data, lost data during electronic upload, incomplete survey data, survey responses with questionable validity, or invalid HIV test results, or because the participant could not be identified as male or female. Reasons for exclusion were not mutually exclusive.

*** Participants were asked whether they “currently have health insurance or health care coverage.” Health insurance was defined for participants as “health plans people get through employment or purchased directly, as well as government programs like Medicare and Medicaid that provide medical care or help pay medical bills.”

††† Additional information available at http://www.whitehouse.gov/administration/eop/onap.
